# Oral Cancer Numerical Index (OCNI): Development and Validation of a Cytology-Based Risk Assessment for Oral Lesions

**DOI:** 10.3390/jcm15124692

**Published:** 2026-06-17

**Authors:** Michael P. McRae, Nadarajah Vigneswaran, Alexander Ross Kerr, Spencer W. Redding, Martin H. Thornhill, Craig Murdoch, Paul M. Speight, Rachelle Wolk, Kritika S. Rajsri, Pooja Gaikwad, Nancy Ruel, Nicolaos J. Christodoulides, John T. McDevitt

**Affiliations:** 1 Custom DX Solutions LLC, Houston, TX 77005, USA; 2 Department of Diagnostic and Biomedical Sciences, The University of Texas Health Science Center at Houston, Houston, TX 77054, USA; 3 Department of Oral and Maxillofacial Pathology, Radiology & Medicine, New York University College of Dentistry, New York, NY 10010, USA; 4 Department of Comprehensive Dentistry and Mays Cancer Center, The University of Texas at San Antonio, San Antonio, TX 78229, USA; 5 Department of Oral & Maxillofacial Medicine, Surgery and Pathology, School of Clinical Dentistry, University of Sheffield, Sheffield S10 2TA, UK; 6 Department of Molecular Pathobiology, Division of Biomaterials, Bioengineering Institute, New York University College of Dentistry, New York, NY 10010, USA

**Keywords:** oral potentially malignant disorders, oral epithelial dysplasia, oral squamous cell carcinoma, cytology, deep learning, artificial intelligence, intelligent cytology microfluidics

## Abstract

**Background/Objectives:** Oral potentially malignant disorders (OPMDs) require accurate risk stratification to identify patients at the highest risk for severe oral epithelial dysplasia (OED) or oral squamous cell carcinoma (OSCC). We developed and internally validated the oral cancer numerical index (OCNI), a quantitative risk score derived from clinical features and deep learning-based brush cytology measurements. **Methods:** This retrospective model development and internal validation study was conducted using data from the multicenter Grand Opportunity study. Prospectively recruited subjects with OPMD with complete data were divided at the subject level into a training set (*n* = 384) and a holdout test set (*n* = 164) using a 70:30 diagnosis-stratified split. The primary endpoint was severe OED or OSCC versus benign diagnoses, and mild and moderate OED. Predictors included age, sex, tobacco history, lesion color, lesion size, multiple lesions, ulcerative morphology, and the percentages of differentiated squamous epithelial and small round cells derived from deep learning-based cytology. Prespecified rule-out and rule-in thresholds were selected in the training set to target 90% sensitivity and 90% specificity, respectively, and then applied to the holdout test set. **Results:** At the prespecified rule-out threshold (OCNI ≤ 37.6), sensitivity was 92% and negative predictive value was 97%. At the rule-in threshold (OCNI > 60.0), specificity was 89% and positive predictive value was 67%. Calibration was good in the holdout set (intercept, −0.07; slope, 1.13; Hosmer–Lemeshow *p* = 0.36), and OCNI increased significantly with worsening histopathologic severity. **Conclusions:** OCNI provided an objective, clinically interpretable estimate of risk for severe OED or OSCC, with strong rule-out and rule-in performance and good calibration. These findings support further external validation of OCNI as an adjunctive tool for oral lesion risk stratification.

## 1. Introduction

Globally, oral potentially malignant disorders (OPMDs) affect approximately 4.7% of individuals, with lower prevalence reported in North America (0.7%), although regional estimates vary substantially across studies [1]. OPMDs may harbor oral epithelial dysplasia (OED) or oral squamous cell carcinoma (OSCC), yet conventional visual and tactile examination (CVTE) alone does not reliably distinguish the risk profile of OPMD [2]. In routine practice, such as in a general dental office, management therefore depends heavily on CVTE, referral, and scalpel biopsy. This creates two important gaps in care. First, it is often difficult to determine which lesions warrant immediate biopsy because lesion color, morphology, and other clinical features overlap substantially across benign and high-risk conditions. Second, there is no widely available objective measure of lesion severity that can support consistent risk stratification and longitudinal monitoring. These limitations may contribute to delayed diagnosis, unnecessary biopsy, and variability in clinical management.

Brush cytology offers a noninvasive adjunct for evaluation of oral lesions [3,4,5], but conventional cytology workflows have been limited by subjective interpretation and inconvenient remote processing. Other adjunctive approaches, including autofluorescence, vital staining, and salivary or molecular biomarkers, are less accurate than cytology [4]. Recent advances in quantitative cytology now enable objective cellular measurements from oral brush samples that correlate with dysplasia and malignancy risk. In the multicenter Grand Opportunity (GO) study, paired clinical, cytologic, and histopathologic data were collected from a large cohort of subjects with suspicious oral lesions [6,7], and deep learning-based cytology analysis identified reproducible changes in cellular phenotypes across the spectrum of disease severity [8].

The Oral Cancer Numerical Index (OCNI) was developed to translate these findings into a clinically actionable risk score. The OCNI integrates clinical lesion characteristics and deep learning-derived cytology measurements to estimate the probability of severe OED or OSCC. The intended advantage of OCNI is to supplement the conventional clinical examination through objective evaluation of oral lesions of uncertain significance. Since OCNI is a continuous, interpretable risk score rather than a qualitative or binary adjunctive finding, different operating thresholds can be selected for various clinical priorities, such as ruling out severe OED or OSCC in lower-prevalence settings or ruling in high-risk disease when expedited biopsy is warranted. The intended clinical application includes two important elements: (1) to support objective risk stratification of OPMDs by identifying lesions at sufficiently low risk to avoid unnecessary referral and/or scalpel biopsy, and (2) to identify high-risk lesions that may warrant expedited biopsy, escalation of care, and/or closer clinical surveillance. The aim of this study was to develop and internally validate the OCNI in subjects with OPMDs and evaluate its discrimination, calibration, and clinically relevant rule-out and rule-in performance for severe OED/OSCC.

## 2. Methods

### 2.1. Study Design and Participants

This retrospective prediction model development and internal validation analysis utilized data from the GO study, a four-site international prospective study that collected paired clinical, cytologic, and histopathologic data from subjects with OPMDs and OSCC [6].

Histopathological and brush cytological samples were collected between July 2010 and December 2012 from three groups: Group 1, subjects with OPMDs who underwent scalpel biopsy as per standard of care; Group 2, subjects with recently diagnosed OSCC; and Group 3, healthy controls without lesions. Group 1 subjects were adults with lesions ≥ 5 mm in diameter with OPMD diagnosed clinically, for which a conventional scalpel biopsy was indicated. Group 2 subjects were adults with a malignant oral lesion confirmed by incisional scalpel biopsy, awaiting definitive treatment, and with the remaining lesion large enough to allow brushing. Group 3 subjects were adults with normal-appearing oral mucosa upon expert clinical examination.

Scalpel biopsy and histopathology were performed in all subjects in Groups 1 and 2. Histopathologic diagnoses comprised benign, mild OED, moderate OED, severe OED/carcinoma in situ (reported in aggregate henceforth as “severe OED”), and OSCC [9]. Histopathologic classification was supported by a two- or three-stage adjudication process involving independent review by oral pathologists [6]. Two consecutive serial histologic sections were prepared and scored by two pathologists blinded to the clinical and microscopic diagnosis and site of the lesion. Upon disagreement in scoring, a third independent pathologist reviewed both sections. This adjudicator was independent of the previous review stages and blinded to the clinical details, original diagnosis, and opinions of the previous reviewers.

For the present OCNI analysis, model development was restricted to subjects with OPMDs to match the intended use population. Healthy controls were subjects without lesions who did not undergo scalpel biopsy and histopathology. Data from healthy controls were used for descriptive and reference analyses but were excluded from model training.

Primary care and general dental practice settings were not included in the study design because the initial objective was to establish the diagnostic accuracy of the OCNI relative to adjudicated histopathologic diagnoses. This design reduces uncertainty in reference-standard assignment but limits direct generalizability to lower-prevalence primary care settings, where OED/OSCC prevalence, operator expertise, and predictive values may differ.

The study was conducted according to the Declaration of Helsinki and was approved by the Institutional Review Boards of participating institutions (University of Texas Health Science Center at San Antonio; University of Texas Health Science Center at Houston; University of Sheffield; Rice University; and New York University). All subjects provided written informed consent.

### 2.2. Study Procedures

Brush cytology collection, specimen processing, microfluidic assay procedures, fluorescence staining, and image acquisition were performed as previously described [7]. In brief, oral brush specimens were obtained from lesions in subjects with OPMDs or OSCC, and from normal-appearing mucosa in healthy controls. Cells were stained with phalloidin–AlexaFluor-647 (#A22287; Life Technologies, Carlsbad, CA, USA) for cytoplasmic visualization and DAPI (#D3571; Life Technologies) for nuclear visualization and imaged automatically using a motorized fluorescence microscope (BX-RFAA; Olympus, Tokyo, Japan) at 10× magnification. For each sample, 25 unique image fields spanning approximately 20 mm^2^ were acquired at three focal planes and combined into enhanced depth-of-field images. These image data served as the basis for deep learning-based cell phenotype detection and for downstream cytology measurements used as candidate OCNI inputs.

### 2.3. Deep Learning-Derived Cytology Measurements

A deep learning object-detection model was trained to identify four oral cytology phenotypes: differentiated squamous epithelial (DSE) cells, small round (SR) cells, leukocytes, and lone nuclei (LN) [8]. The DL object detection model was developed using transfer learning of a YOLOv8 (Ultralytics, Los Angeles, CA, USA) pre-trained model (COCO, large). Bounding-box detections from that model were used to quantify the relative proportions of these phenotypes within each sample. The proportions of DSE cells and SR cells were strongly associated with histopathologic severity and were among the cytologic variables considered for OCNI derivation. The present study did not retrain the object detector, but rather it used locked deep learning-derived cytology measurements as candidate predictors for OCNI.

The reliability and repeatability of the deep learning model were reported elsewhere and are summarized below [8]. Up to six repeat cytology measurements were conducted across 692 subjects, representing a total of 4028 repeat tests. Among all subjects, within-sample reliability was high for all cell phenotype measurements, with reliability by intra-class correlation coefficient (ICC) ranging from 0.87 to 0.98. In terms of repeatability, OCNI demonstrated low variability (%CV 8.2%), and Bland–Altman analysis revealed a mean bias of 0 and limits of agreement (±1.96 SD) of ±7.2.

### 2.4. Model Development and Validation

The OCNI model was developed using penalized logistic regression. The model’s primary endpoint was the discrimination of benign, mild OED, and moderate OED versus severe OED or worse (denoted 2,3,4|5,6). Candidate predictors were selected a priori based on clinical and cytologic evidence and included age, sex, tobacco history, lesion color, lesion size, multiple lesions, ulcerated lesion appearance, percentage of DSE cells, and percentage of SR cells. Tobacco history was defined as satisfying one or more of the following: at least 100 cigarettes in lifetime, 20 cigars or pipes in lifetime, or use of chewing tobacco or snuff for more than 1 year in lifetime. Lesion color was modeled as an ordinal clinical variable reflecting malignancy risk (white < red < mixed red and white). Lesion size was coded from clinical measurements, with large size defined as major axis length at least 10 mm or diffuse/unmeasurable extent. Cytologic predictors were obtained from the deep learning cell phenotype classifier described in the previous section [8].

All eligible lesion cases with complete predictor and outcome data were randomly divided at the subject level into development and holdout test sets using a 70:30 split, with stratification by histopathologic diagnosis to preserve representation of the diagnostic categories across partitions. Within the training set, predictor selection and coefficient shrinkage were performed using least absolute shrinkage and selection operator (LASSO) logistic regression with 10-fold cross-validation. Predictors were standardized internally during penalized model fitting so that the lasso penalty was applied on a common scale; however, the final coefficients were presented on the original predictor scale and were therefore interpreted in the native units of each variable. The penalty parameter was chosen by minimum deviance. Continuous model output was transformed to a 0–100 scale and designated the OCNI, with higher values indicating a higher predicted probability of severe OED or OSCC. The final locked model, including intercept and regression coefficients selected from the cross-validated LASSO procedure, was then applied to the holdout test set without re-estimation.

Several design features were used to control potential overfitting: diagnosis-stratified subject-level splitting, a priori limitation of candidate predictors, LASSO shrinkage with 10-fold cross-validation in the training set, pre-specification of operating thresholds in the training set only, application of a locked model to the holdout internal test set, and calibration assessment using the holdout predictions. Histopathologic adjudication was performed independently of the OCNI prediction model, and OCNI threshold performance was calculated after the model was locked.

A continuous risk model can be applied using distinct rule-out and rule-in thresholds that reflect different clinical priorities. A rule-out threshold prioritizes high sensitivity, minimizing false-negative results and supporting use in lower-prevalence primary care settings where the goal is to capture most patients with severe OED/OSCC who require referral or biopsy. Conversely, a rule-in threshold prioritizes high specificity, minimizing false-positive results and supporting use in higher-risk surveillance or specialist settings where the goal is to identify patients most likely to warrant expedited biopsy or intensified follow-up. These thresholds, therefore, represent complementary operating points of the same model, aligned with different disease prevalence, clinical settings, and tolerances for missed disease versus unnecessary intervention.

Prespecified operating thresholds for converting the continuous OCNI score into categorical risk groups were determined from the training set based on diagnostic sensitivity and specificity. For the rule-out model, the OCNI cutoff was selected for 90% sensitivity on the training set. For the rule-in model, the OCNI cutoff was selected for 90% specificity on the training set. These prespecified cut points were then applied to the holdout test set for evaluation of sensitivity, specificity, predictive values, and likelihood ratios. Threshold derivation was therefore confined to the training set, whereas test-set performance was used for independent evaluation.

One subject with a missing age value was imputed using simple median imputation; sensitivity analyses confirmed that classification relative to the OCNI decision thresholds remained unchanged when age was varied from 18 to 96 years. No other missing data were present for model development variables, and no additional imputation was required.

Internal validation was performed in the independent subject-level holdout test set. Model discrimination was summarized by the area under the receiver operating characteristic curve (AUROC), with bootstrap resampling used to estimate 95% confidence intervals. For threshold-based analyses, sensitivity, specificity, positive predictive value (PPV), negative predictive value (NPV), positive likelihood ratio (PLR), and negative likelihood ratio (NLR) and their 95% confidence intervals were calculated from the test-set 2 × 2 contingency table.

In addition to binary classification performance, the continuous OCNI score was examined across ordered histopathologic categories using grouped distribution plots and ordinal trends. Because the test set was fully separated from the training set at the subject level, these analyses provided an independent estimate of generalization performance.

Calibration was assessed in the held-out test set by comparing predicted and observed event probabilities. Overall calibration was summarized using the intercept and slope obtained from logistic recalibration models, and overall prediction error was summarized using the Brier score. Calibration was additionally assessed using the Hosmer-Lemeshow goodness-of-fit test, in which subjects were grouped into 5 approximately equal-sized risk strata based on predicted probability, and observed versus expected event counts were compared using chi-square. Calibration plots were constructed by grouping subjects according to predicted risk and plotting mean predicted probability against observed event rate with exact binomial confidence intervals, supplemented by a smoothed calibration curve estimated using local logistic regression. Bootstrap resampling was used to derive confidence bands for the smoothed calibration curve.

### 2.5. Statistical Analysis

Descriptive statistics (mean ± SD or n [%]) were summarized for healthy controls, subjects with benign diagnoses, and subjects with OED or OSCC. Comparisons between subjects with benign diagnoses and those with OED or OSCC were conducted using an independent two-sample *t*-test for continuous variables and a chi-squared test for categorical variables, with *p* ≤ 0.05 considered statistically significant.

Univariable and multivariable logistic regression analyses were performed to evaluate associations of clinical and cytological predictors with severe OED/OSCC and to determine whether cytological variables retained independent associations after adjustment for clinical variables.

The following cytological test parameters were calculated: relative percentages of DSE cells, SR cells, and leukocytes; approximate median cell diameter of DSE and SR cells (i.e., square root of bounding box area converted from pixels to μm); and the OCNI [7], which is a score from 0 to 100 that represents the probability of OED or OSCC (predictors included age, sex, tobacco history, lesion color, lesion size, lesion appearance, presence of multiple lesions, DSE cells, and SR cells).

OCNI values in the holdout test set were summarized across ordered histopathologic categories (benign; mild, moderate, and severe OED; and malignant) using the median and interquartile range. Differences across groups were evaluated with the Kruskal–Wallis test, and monotonic trend across increasing histopathologic severity was assessed using the Jonckheere–Terpstra test.

The null hypothesis was that OCNI would not discriminate severe OED/OSCC from benign, mild OED, or moderate OED beyond chance and would not show a graded association with histopathologic severity.

Reference limits for cytology test parameters were established from 144 healthy controls by estimating one-sided lower or upper reference limits as the 5th or 95th percentile of the reference distribution.

All statistical analyses and figure generation for OCNI development and validation were performed in MATLAB R2025b (Natick, MA, USA).

## 3. Results

### 3.1. Subject Characteristics

**Figure 1:**
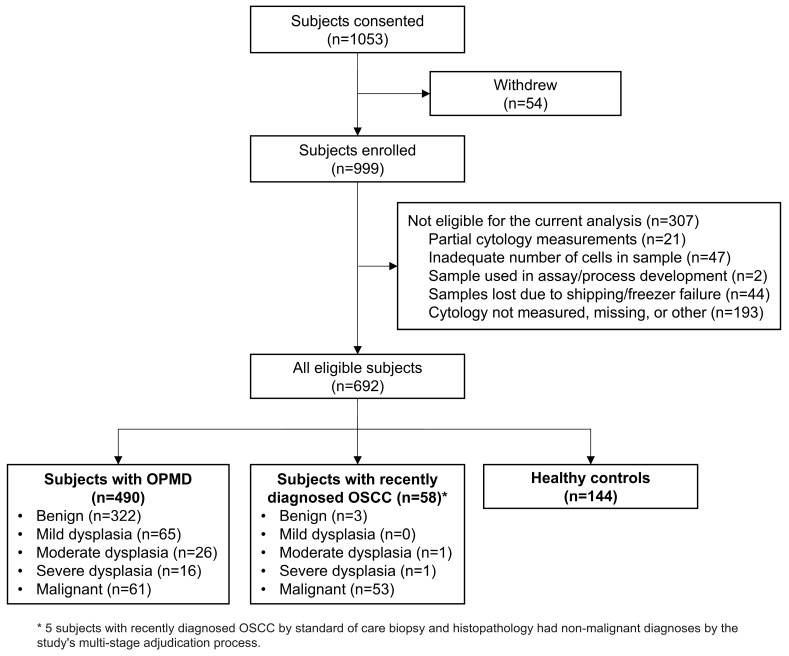
Subject disposition for the Grand Opportunity Study.

Of the 1053 subjects enrolled in the GO study, 54 withdrew, and 307 were excluded due to invalid measurements, lost or missing samples, or results not analyzed because of funding constraints (Figure 1). A total of 692 subjects were included in this analysis: 144 healthy controls, 325 benign, 65 mild OED, 27 moderate OED, 17 severe OED, and 114 OSCC. We previously reported results for 486 subjects [7]. In this current analysis, data from an additional 206 subjects, which had been collected but not analyzed, were included for either training or test set evaluation.

Table 1 summarizes subject characteristics and cytology test parameters in healthy controls, subjects with benign or mild–moderate OED, and subjects with severe OED or OSCC. Compared with the benign/mild–moderate OED group, subjects with severe OED/OSCC were older (60 ± 12 vs. 56 ± 14 years, *p* = 0.0152) and more often male (66% vs. 46%, *p* = 0.0001). Tobacco exposure was also more common in the severe OED/OSCC group, including any tobacco history (73% vs. 59%, *p* = 0.0033), former smoking (70% vs. 55%, *p* = 0.0044), current smoking (37% vs. 23%, *p* = 0.0036), and greater smoking pack-years (20 ± 25 vs. 12 ± 20, *p* < 0.0001). Alcohol-related variables did not differ significantly between groups.

Lesion characteristics differed substantially by histopathologic severity. Severe OED/OSCC lesions were larger overall (26 ± 16 vs. 16 ± 12 mm, *p* < 0.0001) and were less likely to be diffuse or unmeasurable (6% vs. 14%, *p* = 0.0292). Severe OED/OSCC was associated with a lower frequency of white lesions (12% vs. 45%, *p* < 0.0001) and higher frequencies of red lesions (25% vs. 15%, *p* = 0.0145) and mixed red-and-white lesions (63% vs. 40%, *p* < 0.0001). In addition, severe OED/OSCC lesions more commonly presented as nodules or masses (51% vs. 18%, *p* < 0.0001), ulcers (47% vs. 10%, *p* < 0.0001), and erosive lesions (16% vs. 7%, *p* = 0.0058), while patch/plaque morphology (32% vs. 74%, *p* < 0.0001) and multiple lesions (23% vs. 45%, *p* < 0.0001) were less common.

### 3.2. Cytology Test Parameters

Cytology test parameters demonstrated significant differences across groups, with a progressive shift away from the healthy control profile as disease severity increased. Mean DSE cell percentage decreased from 96 ± 3% in controls to 83 ± 17% in benign/mild–moderate OED and 46 ± 31% in severe OED/OSCC (*p* < 0.0001). In contrast, SR cell percentage increased from 3 ± 2% in controls to 11 ± 10% and 22 ± 14%, respectively (*p* < 0.0001), and leukocyte percentage increased from 0.9 ± 0.7% to 7 ± 13% and 32 ± 28% (*p* < 0.0001). Median cell diameter similarly decreased with severity, from 72 ± 4 μm in controls to 68 ± 8 μm in benign/mild–moderate OED and 55 ± 13 μm in severe OED/OSCC (*p* < 0.0001). Overall, these findings indicate that severe OED/OSCC is associated with distinct clinical and cytologic features compared with lower-risk lesions.

Univariable analysis showed that all clinical and cytological variables were significantly associated with severe OED/OSCC (Supplemental Table S1), and multivariable models demonstrated that both clinical and cytological variables retained independent associations with severe OED and OSCC (Supplemental Table S2).

### 3.3. Diagnostic Performance

The final LASSO logistic regression model retained the following predictors: age, sex, tobacco history, lesion color, lesion size, multiple lesions, ulcerative lesion morphology, DSE cell percentage, and SR cell percentage (Table 2). The retained coefficients indicated the expected directional contributions: higher age, male sex, tobacco history, higher-risk lesion color, larger lesion size, ulcerative morphology, and higher SR cell percentage increased the predicted probability of severe OED/OSCC, whereas higher DSE cell percentage and lesion multiplicity decreased the predicted probability. Because Table 2 presents coefficients on the original predictor scale, coefficient magnitude should not be interpreted as a direct ranking of variable importance across predictors measured in different units.

In the independent holdout test set (*n* = 164), the OCNI demonstrated strong performance for both rule-out and rule-in stratification of severe OED/OSCC (Table 3) with an AUROC of 0.92 (0.83–0.96). At the prespecified rule-out threshold of OCNI ≤ 37.6, sensitivity was 92% (95% CI, 79–98%), and NPV was 97% (91–99%). Only 3 severe OED/OSCC cases were missed. In a descriptive subgroup analysis, the rule-out threshold identified 33 of 34 OSCC cases (97.1%; exact 95% CI, 84.7–99.9%) and 3 of 5 severe OED cases (60.0%; exact 95% CI, 14.7–94.7%) in the holdout set; the severe OED subgroup estimate should be interpreted cautiously because of the small denominator. If in standard general dental practice all subjects with OPMD had been recommended for scalpel biopsy, the use of OCNI ≤ 37.6 would have potentially rendered 93 (57%) scalpel biopsies unnecessary. At the rule-in threshold of OCNI > 60.0, specificity was 89% (82–94%), and PPV was 67% (51–80%), indicating good ability to identify lesions at high risk for severe OED/OSCC.

The holdout test set also demonstrated similar diagnostic performance to the training set, supporting the internal consistency of the model and suggesting that overfitting is unlikely.

Calibration analysis in the holdout test set demonstrated good agreement between predicted and observed risk. The calibration intercept was close to 0 (−0.07, 95% CI −0.42 to 0.29), indicating little evidence of systematic over- or underestimation, and the calibration slope was close to 1 (1.13, 95% CI 0.74 to 1.51), indicating no substantial evidence of overfitting. There was no evidence of lack of fit by the Hosmer-Lemeshow test (*p* = 0.3643). Grouped calibration plots showed an overall increase in observed event rates across increasing predicted-risk strata, although some variability was present within individual groups due to small subgroup sizes (Supplemental Figure S1).

### 3.4. Distribution of OCNI

**Figure 2:**
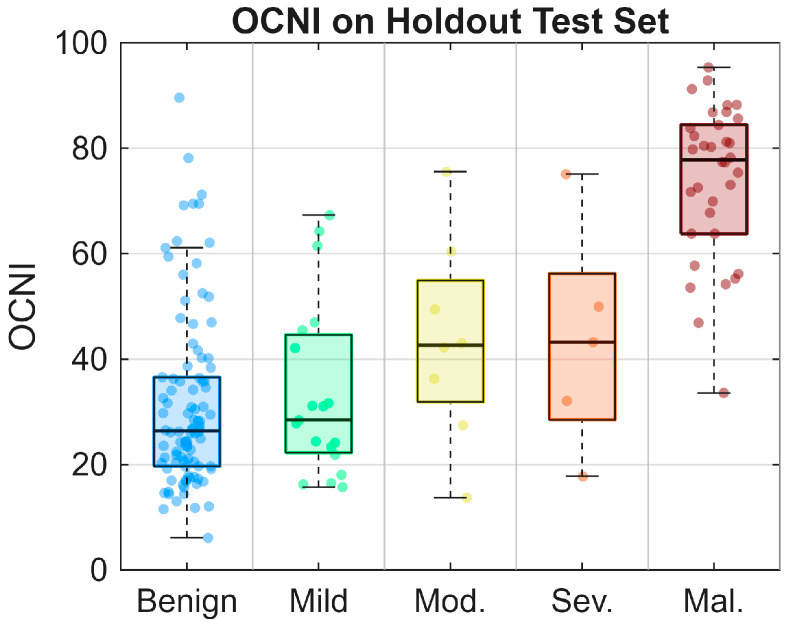
Distribution of oral cancer numerical index (OCNI) values in the holdout test set by histopathologic diagnosis. Individual subject scores are shown as jittered points, with boxplots summarizing the median, interquartile range, and overall spread within each diagnostic category. OCNI values were lowest in benign lesions and increased progressively across mild, moderate, severe dysplasia, and malignant lesions, consistent with increasing histopathologic severity.

Figure 2 depicts the distribution of OCNI values for subjects in the holdout test set by histopathology. OCNI increased progressively with worsening histopathologic severity, with median (IQR) values of 26.4 (19.7–36.6) for benign lesions, 28.5 (22.3–44.6) for mild OED, 42.6 (31.9–54.9) for moderate OED, 43.2 (28.5–56.2) for severe OED, and 77.8 (63.8–84.4) for malignant lesions (Supplemental Table S3). Overall differences across histopathologic categories were highly significant (Kruskal–Wallis *p* < 0.0001), and there was a strong monotonic increase in OCNI across the ordered categories from benign to malignant disease (Jonckheere–Terpstra *p* < 0.0001). These findings support a graded relationship between OCNI and histopathologic severity, with the highest scores observed in malignant lesions and intermediate values in dysplastic lesions.

**Figure 3:**
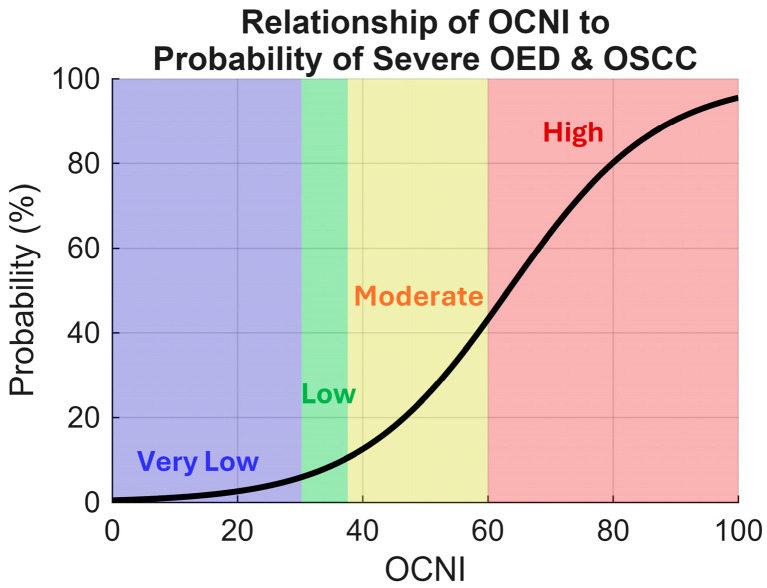
Relationship between the oral cancer numerical index (OCNI) and predicted probability of severe oral epithelial dysplasia (OED) or oral squamous cell carcinoma (OSCC). The solid black curve shows the modeled probability of severe OED or OSCC across the continuous OCNI scale. Background shading defines four OCNI-based risk strata: very low risk (<30.2), low risk (≤37.6), moderate risk (37.6–60), and high risk (>60). These zones provide a framework for translating the continuous OCNI score into clinically interpretable categories of increasing disease risk.

Figure 3 illustrates the logistic relationship between OCNI and the predicted probability of severe OED/OSCC, showing a progressive increase in risk with higher OCNI values. The probability remained very low for values below the healthy control reference cutoff of 30.2 (<6%), increased modestly at the rule-out cutoff of 37.6 (10.5%), and rose substantially at the rule-in cutoff of 60.0 (43%). These thresholds support the definition of clinically meaningful risk zones.

### 3.5. Reference Limits in Healthy Controls

In healthy controls, cytology test parameters showed relatively narrow distributions (Table 4). DSE cell percentage was high in controls, with a median of 96.5%, yielding a lower reference limit of >90.7%. Parameters expected to remain low in healthy subjects showed correspondingly low reference limits, including SR cells (median 2.7%; reference limit < 7.5%), leukocytes (median 0.7%; reference limit < 2.0%), and OCNI (median 14.5; reference limit < 30.2). Median cell diameter was relatively stable in controls, with a median of 72.4 μm and a lower reference limit of >66.1 μm. Overall, these findings define the expected range of cytological measurements in healthy individuals and provide reference thresholds against which abnormal test results may be interpreted.

## 4. Discussion

In this study, we developed and internally validated the OCNI, a multimodal model that integrates clinical lesion features with deep learning-derived oral cytology measurements to estimate the probability of severe OED or OSCC in patients with OPMD. The principal findings are threefold. First, OCNI demonstrated strong performance in an independent holdout test set for both low-risk and high-risk stratification, with high sensitivity and NPV at the rule-out threshold and high specificity and PPV at the rule-in threshold. Second, the model showed good calibration, with predicted risks closely aligned with observed outcomes. Third, OCNI increased progressively across histopathologic categories, supporting its validity as a continuous measure of disease severity rather than a simple binary classifier. Collectively, these findings indicate that combining quantitative cytology with clinical lesion characteristics can provide an objective and clinically interpretable estimate of oral cancer risk.

The main implication of these findings is that OCNI may serve as a useful adjunct for risk stratification of OPMDs. Its intended role is not to replace biopsy or histopathology, but to aid decision-making in settings where lesion appearance alone may be insufficient to guide management confidently. In this framework, a low OCNI score may help rule out lesions with severe OED/OSCC to aid in the decision to avoid scalpel biopsy. In contrast, a high score may help rule in lesions with severe OED/OSCC to warrant expedited biopsy, referral, or closer surveillance. The graded increase in OCNI across histopathologic severity further suggests that the score captures biologically relevant variation across the spectrum of disease. This feature may be important for future longitudinal applications. A prospective longitudinal study of malignant transformation in subjects with OED and cancer recurrence in subjects with prior OSCC is underway.

The present study builds on a substantial body of prior work in quantitative oral cytology. A prospective validation study established one of the largest oral cytology databases in OPMD, with cytologic measurements correlated to multiple histopathologic categories [6]. Earlier versions of the cytology-on-a-chip platform, using conventional feature extraction and machine learning, achieved diagnostic performance comparable to or better than several commercially available adjuncts [10,11,12]. The subsequent development of a brush cytology collection kit, disposable microfluidic cartridges, and integrated instrumentation enabled point-of-care implementation. A deep learning-based cell phenotype classifier further improved the cytologic measurements, thereafter referred to as the intelligent cytology microfluidics (Cyt-MF) system, and demonstrated excellent within-individual reproducibility [8]. The present work extends this platform by incorporating the deep learning-based cytology platform into a clinically interpretable numerical risk model. In this sense, the Cyt-MF system represents a logical progression from quantitative cytology toward point-of-care, expert-level risk stratification for OED and OSCC.

Other adjunctive approaches which include cytologic testing platforms, autofluorescence devices, tissue reflectance systems, vital staining, and salivary biomarker assays have been studied in patients with visible oral mucosal abnormalities; however, performance has been mixed, with independent evaluations highlighting concerns regarding low or variable specificity, particularly when distinguishing OED or malignancy from benign, inflammatory, or reactive lesions [4,5,13,14,15,16]. Blood-based methylation assays such as Galleri have been validated in pan-cancer cohorts and asymptomatic screening populations rather than in patients presenting with suspicious oral lesions [17,18,19]. In a meta-analysis of adjuncts, cytologic testing had the highest accuracy [4]. Critically, none of these approaches have been validated in the intended population encompassing the full OPMD spectrum, which includes approximately 15-fold more OED than OSCC, and OED inclusion substantially erodes diagnostic accuracy [7]. AI-linked cytologic analysis, as reported here, is positioned to address these gaps: removing OED subjects in silico yields AUC values of 0.97 to 0.99 for healthy-versus-OSCC, while retaining the full prospective population decreases AUC to 0.92.

In contrast, the principal strength of this Cyt-MF approach is the close alignment between the methodological design and the intended clinical application. The model was derived from a multicenter prospective study of patients undergoing evaluation for oral lesions and was trained specifically in the intended-use population of subjects with OPMD rather than in an extreme case–control comparison of established cancer versus healthy controls. This design captured a clinically meaningful spectrum of disease severity, including OPMDs with benign diagnoses, OPMDs harboring the full spectrum of dysplasia grading (mild, moderate, and severe), and OSCC, thereby addressing the central clinical question in oral lesion management: whether a suspicious lesion is sufficiently low risk to defer biopsy or sufficiently high risk to warrant expedited biopsy. Additional methodological strengths include two- or three-stage histopathologic adjudication, subject-level separation of development and holdout test sets with diagnosis-stratified sampling, prespecified rule-out and rule-in thresholds derived exclusively in the training set, and independent assessment of both discrimination and calibration in the holdout cohort. The resulting framework, therefore, extends beyond simple binary detection and instead provides a calibrated and clinically actionable estimate of risk across the OPMD disease spectrum.

The dichotomous endpoint of severe OED versus benign, mild, and moderate OED was selected based on the progressive increase in risk for malignant transformation with increasing OED severity. Previous research articles and systematic reviews reported malignant transformation rates of 0–8% for mild dysplasia, 3–18% for moderate dysplasia, and 16–40% for severe dysplasia [20,21,22,23,24]. Therefore, we considered severe OED to be the most appropriate high-risk preinvasive category for the model endpoint given its substantially higher rate of malignant transformation relative to mild and moderate OED. This should be viewed as a conservative modeling choice for immediate risk stratification rather than implying that moderate OED is low risk or does not warrant ongoing surveillance.

Referral patterns in general dental practice studies help clarify the practical setting in which OCNI may be used. In a recent National Dental Practice-Based Research Network study [25], 65% of U.S. general dental practitioners reported referring patients with suspicious oral lesions for consultation or biopsy, 87% of those referrals were directed to oral and maxillofacial surgeons, and only 22% reported personally performing biopsies. Referrals were usually accompanied by lesion location, signs or symptoms, and lesion history, and biopsy results were communicated back in writing in more than 95% of cases [25]. These findings suggest that suspicious lesions in general dental practice commonly enter a referral-based management pathway rather than an in-office biopsy pathway. In that setting, OCNI may be particularly useful as an objective adjunct to help general dentists prioritize referral urgency.

**Figure 4:**
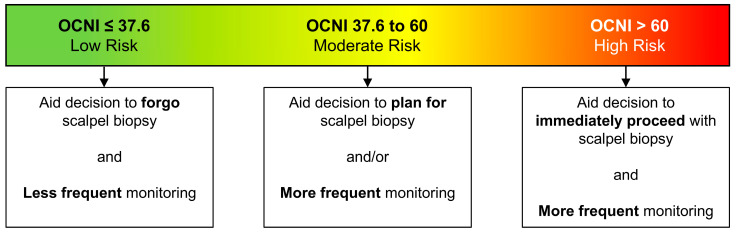
Proposed algorithm for implementation of the oral cancer numerical index (OCNI) in clinical practice.

OCNI-based risk zones were defined to provide an objective framework for clinical management (Figure 4). Scores ≤ 30.2 fell within the upper reference limit established from healthy controls with clinically normal mucosa and indicated very low risk. Notably, the median OCNI value in mild OED was 29, further supporting this threshold as consistent with low-risk disease. Scores ≤ 37.6 were classified as low risk for severe OED/OSCC and may aid the decision to avoid immediate scalpel biopsy and may require less frequent monitoring. Scores > 37.6 to ≤60.0 were classified as moderate risk, warranting consideration for scalpel biopsy based on the overall clinical context and/or more frequent monitoring of the lesion. Scores > 60.0 were classified as high risk for severe OED/OSCC and would support a recommendation for immediate scalpel biopsy and even more frequent monitoring.

This analysis has several limitations. First, model development and validation were performed on data from the same study population, so the results represent internal validation rather than external validation. Second, the study population was derived from patients evaluated in secondary or specialty care settings, where disease prevalence, lesion spectrum, and clinical expertise may differ from those encountered in primary care or general practice dental settings. Predictive values, which are prevalence dependent, may differ in primary care or general dental practice settings from the values reported herein. In lower-prevalence general dental practice settings, PPV would be expected to decrease, and NPV would be expected to remain high or increase. Additional validation in primary care and general practice dental settings is warranted. Third, some histopathologic subgroups, particularly severe OED, were relatively small, which limits precision in subgroup estimates. Fourth, exclusion of subjects with invalid measurements, lost or missing samples, or unanalyzed results may have introduced selection bias, although only subjects with complete predictor and outcome data could be included in the current model-development analysis. Fifth, routine implementation in primary care or general dental practices will require additional evaluation of cost, accessibility, workflow integration, reproducibility across operators and instruments, and clinical impact on biopsy and referral decisions. Finally, OCNI was developed to identify concurrent severe OED/OSCC, and its value for predicting future malignant transformation will be established in an ongoing prospective longitudinal study.

In conclusion, OCNI provides an objective, multimodal estimate of risk for severe OED or OSCC that combines clinical examination findings with deep learning-derived cytology features. The OCNI showed strong discrimination, calibration, and clinically relevant rule-out and rule-in performance, while also tracking increasing histopathologic severity across the disease spectrum. These findings support further external validation of the Cyt-MF system as an adjunctive tool for oral lesion risk stratification.

## Figures and Tables

**Table 1:** Subject characteristics and cytology test parameters by histopathology (benign, mild, and moderate dysplasia versus severe dysplasia and OSCC) and healthy controls.

	Controls (*n* = 144)	Benign and Mild–Moderate OED (*n* = 417)	Severe OED and OSCC (*n* = 131)	*p* Value ^a^
Age (years)	45.8 ± 15.6	56.4 ± 14.0	59.7 ± 12.1	**0.0152**
Male	42 (29.2%)	191 (45.8%)	86 (65.6%)	**0.0001**
Race				
White	129 (89.6%)	352 (84.4%)	106 (80.9%)	0.4196
Black or African American	8 (5.6%)	26 (6.2%)	16 (12.2%)	**0.0398**
Asian	6 (4.2%)	33 (7.9%)	7 (5.3%)	0.4272
American Indian or Alaskan Native	3 (2.1%)	0 (0.0%)	2 (1.5%)	0.0896
Other	2 (1.4%)	8 (1.9%)	3 (2.3%)	1.0000
Ethnicity				
Hispanic	60 (41.7%)	57 (13.7%)	33 (25.2%)	**0.0030**
Alcohol				
History of alcohol	128 (88.9%)	373 (89.4%)	117 (89.3%)	1.0000
Current drinker	82 (56.9%)	271 (65.0%)	79 (60.3%)	0.3849
Days per year	52.9 ± 84.4	91.2 ± 120.5	114.8 ± 144.6	0.0635
Average drinks per day	2.2 ± 3.0	2.1 ± 2.3	2.2 ± 2.8	0.6325
Tobacco				
Any tobacco history	66 (45.8%)	244 (58.5%)	96 (73.3%)	**0.0033**
Former smoker	62 (43.1%)	229 (54.9%)	91 (69.5%)	**0.0044**
Current smoker	28 (19.4%)	97 (23.3%)	48 (36.6%)	**0.0036**
Smoking pack-years	6.1 ± 12.8	11.5 ± 20.2	20.3 ± 24.9	**<0.0001**
Lesion size				
Major axis length (mm)	-	15.9 ± 11.5	25.8 ± 16.3	**<0.0001**
Minor axis length (mm)	-	9.9 ± 7.4	19.5 ± 14.0	**<0.0001**
Diffuse or unable to measure	-	57 (13.7%)	8 (6.1%)	**0.0292**
Lesion color				
White	-	187 (44.8%)	15 (11.5%)	**<0.0001**
Red	-	64 (15.3%)	33 (25.2%)	**0.0145**
Red and white	-	165 (39.6%)	83 (63.4%)	**<0.0001**
Lesion appearance				
Patch/plaque	-	308 (73.9%)	42 (32.1%)	**<0.0001**
Nodule/mass	-	76 (18.2%)	67 (51.1%)	**<0.0001**
Ulcer	-	40 (9.6%)	62 (47.3%)	**<0.0001**
Erosive	-	31 (7.4%)	21 (16.0%)	**0.0058**
Multiple lesions	-	188 (45.1%)	30 (22.9%)	**<0.0001**
Cytology test parameters				
DSE Cells (%)	95.8 ± 2.8	82.7 ± 17.4	45.9 ± 30.7	**<0.0001**
SR Cells (%)	3.3 ± 2.3	10.7 ± 10.1	21.7 ± 14.2	**<0.0001**
Leukocytes (%)	0.9 ± 0.7	6.6 ± 13.3	32.3 ± 28.0	**<0.0001**
Lone nuclei (%)	17.8 ± 14.2	23.0 ± 15.7	17.6 ± 14.0	**0.0005**
Median cell diameter (μm)	72.4 ± 3.6	67.5 ± 7.8	54.6 ± 12.9	**<0.0001**

^a^ *p* values for comparison of benign and mild–moderate OED versus severe OED and OSCC, continuous data by independent two-sample *t*-test and proportions by chi-squared test. Bold values indicate statistical significance (*p* ≤ 0.05). Abbreviations: OED, oral epithelial dysplasia (mild, moderate, severe); OSCC, oral squamous cell carcinoma; DSE, differentiated squamous epithelial cells; SR, small round parabasal-like cells.

**Table 2:** LASSO logistic regression coefficients for the oral cancer numerical index (OCNI) model.

Predictor	Coefficient
Intercept	−1.1356
Age (years)	0.0170
Male sex	0.3594
Tobacco history	0.3572
Lesion color	0.0907
Lesion size	0.5516
Multiple lesions	−0.6616
Ulcerative lesion	0.6622
DSE cells (%)	−0.0181
SR cells (%)	0.0254

**Table 3:** Diagnostic performance (95% CI) of the oral cancer numerical index (OCNI) for identification of severe oral epithelial dysplasia (OED) or oral squamous cell carcinoma (OSCC) in the training and hold-out test sets. Performance is reported for two OCNI thresholds: a lower threshold (≤37.6) intended to support rule-out of severe OED/OSCC and a higher threshold (>60.0) intended to support rule-in of high-risk disease.

	Training Set (*n* = 384)	Holdout Test Set (*n* = 164)
OCNI ≤ 37.6 (rule out)		
Sensitivity (%)	89.1 (80.9–94.7)	92.3 (79.1–98.4)
Specificity (%)	68.2 (62.5–73.5)	72.0 (63.3–79.7)
PPV (%)	46.9 (39.3–54.5)	50.7 (38.6–62.8)
NPV (%)	95.2 (91.4–97.7)	96.8 (90.9–99.3)
PLR	2.80 (2.33–3.36)	3.30 (2.45–4.43)
NLR	0.16 (0.09–0.29)	0.11 (0.04–0.32)
Miss rate, n (%)	10 (10.9%)	3 (7.7%)
OCNI > 60.0 (rule in)		
Sensitivity (%)	67.4 (56.8–76.8)	71.8 (55.1–85.0)
Specificity (%)	92.1 (88.4–94.9)	88.8 (81.9–93.7)
PPV (%)	72.9 (62.2–82.0)	66.7 (50.5–80.4)
NPV (%)	90.0 (86.0–93.1)	91.0 (84.4–95.4)
PLR	8.56 (5.64–12.99)	6.41 (3.77–10.91)
NLR	0.35 (0.26–0.48)	0.32 (0.19–0.53)
Miss rate, n (%)	30 (32.6%)	11 (28.2%)

Abbreviations: PPV, positive predictive value; NPV, negative predictive value; PLR, positive likelihood ratio; NLR, negative likelihood ratio.

**Table 4:** Reference limits of cytology test parameters in healthy controls (*n* = 144).

	Min.	2.5^th^ Percentile	Median	Mean	97.5^th^ Percentile	Max.	SD	Reference Limit
DSE cells (%)	76.5	87.6	96.5	95.8	98.4	98.7	2.8	>90.7
SR cells (%)	0.7	1.1	2.7	3.3	10.1	16.4	2.3	<7.5
Leukocytes (%)	0.1	0.2	0.7	0.9	2.4	7.0	0.7	<2.0
Median cell diameter (μm)	57.9	64.4	72.4	72.4	78.8	81.7	3.6	>66.1
OCNI	7.5	7.8	14.5	15.9	31.6	45.4	6.9	<30.2

Abbreviations: DSE cells, differentiated squamous epithelial cells; SR cells, small round parabasal-like cells; OCNI, oral cancer numerical index.

## Data Availability

Individual patient data will not be shared.

## Supplementary Materials

The following supporting information can be downloaded at: https://www.mdpi.com/article/10.3390/jcm15124692/s1.
