# Supplementary material for: Oral Cancer Numerical Index (OCNI): Development and Validation of a Cytology-Based Risk Assessment for Oral Lesions
Source: J Clin Med. 2026 Jun 17;15(12):4692. doi: 10.3390/jcm15124692 (PMC13301221; doi:10.3390/jcm15124692)
Supplement: Supplementary file 1 [file jcm-15-04692-s001.zip › jcm-4351111-supplementary.pdf]

Supplemental Materials

Supplemental Table S1. Univariable logistic regression analysis of clinical and cytological predictors for the prediction of severe dysplasia and OSCC.

Predictor	OR (95% CI)	P value
Age (years)	1.02 (1.00–1.03)	**0.016**
Male	2.26 (1.50–3.40)	**<0.001**
Tobacco history	1.94 (1.26–3.00)	**0.003**
Lesion color: red vs white	6.46 (3.30–12.67)	**<0.001**
Lesion color: mixed vs white	6.30 (3.50–11.35)	**<0.001**
Lesion size	2.90 (1.67–5.04)	**<0.001**
Multiple lesions	0.36 (0.23–0.57)	**<0.001**
Ulcerative lesion	8.47 (5.28–13.59)	**<0.001**
DSE cells (%)	0.95 (0.94–0.96)	**<0.001**
SR cells (%)	1.07 (1.06–1.09)	**<0.001**

Supplemental Table S2. Multivariable logistic regression analysis of clinical and cytological predictors for the prediction of severe dysplasia and OSCC.

Predictor	OR (95% CI)	P value
**Clinical variables only**		
Age (years)	1.03 (1.01-1.05)	**0.004**
Male	3.11 (1.82-5.31)	**<0.001**
Tobacco history	1.96 (1.13-3.39)	**0.016**
Lesion color: red vs white	7.12 (3.21-15.80)	**<0.001**
Lesion color: mixed vs white	7.60 (3.83-15.10)	**<0.001**
Lesion size	4.03 (2.08-7.81)	**<0.001**
Multiple lesions	0.22 (0.13-0.39)	**<0.001**
Ulcerative lesion	5.41 (3.07-9.53)	**<0.001**
**Cytology variables only**		
DSE cells (%)	0.95 (0.94-0.96)	**<0.001**
SR cells (%)	1.01 (0.99-1.04)	0.300
**Clinical and cytology variables**		
Age (years)	1.03 (1.01-1.06)	**0.002**
Male	2.96 (1.63-5.39)	**<0.001**
Tobacco history	1.79 (0.96-3.32)	0.066
Lesion color: red vs white	2.43 (0.97-6.07)	0.058
Lesion color: mixed vs white	3.48 (1.67-7.26)	**<0.001**
Lesion size	2.52 (1.20-5.30)	**0.015**
Multiple lesions	0.28 (0.15-0.52)	**<0.001**
Ulcerative lesion	3.38 (1.80-6.38)	**<0.001**
DSE cells (%)	0.96 (0.95-0.97)	**<0.001**
SR cells (%)	1.01 (0.99-1.04)	0.351

Supplemental Table S3. Median and interquartile ranges for OCNI on the holdout test set by histopathologic diagnosis

Histopathologic Diagnosis	n	Median (IQR)
Benign	98	26.4 (19.7–36.6)
Mild	19	28.5 (22.3–44.6)
Moderate	8	42.6 (31.9–54.9)
Severe	5	43.2 (28.5–56.2)
Malignant	34	77.8 (63.8–84.4)

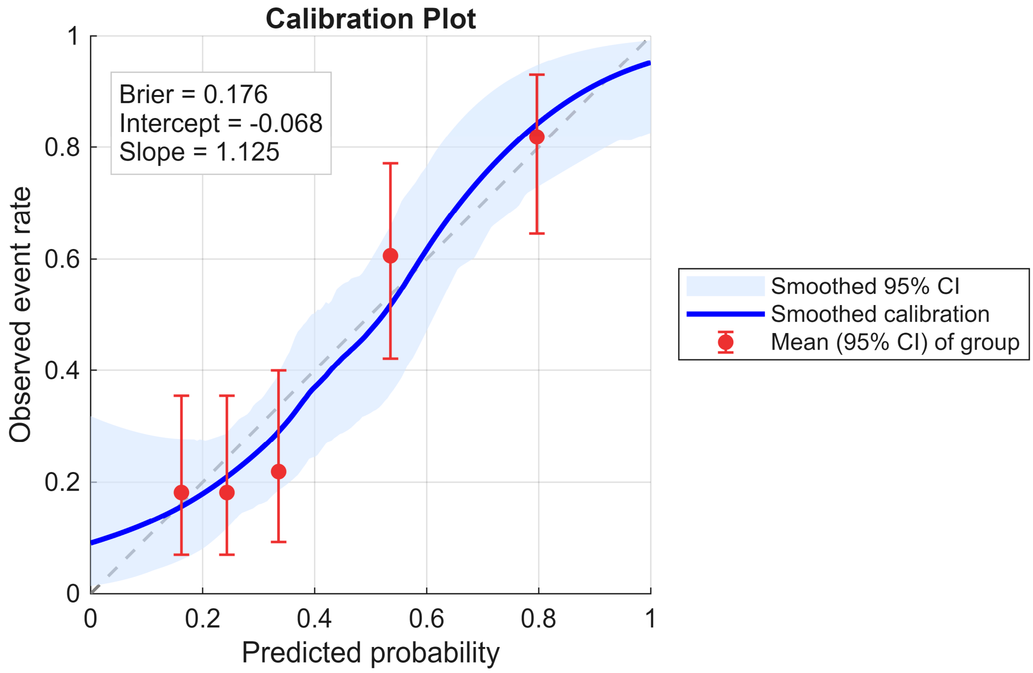

Supplemental Figure S1. Calibration of the Oral Cancer Numerical Index (OCNI) in the hold-out test set. The dashed gray line indicates ideal calibration, where predicted probability equals observed event rate. The solid blue line shows the smoothed calibration curve, and the shaded blue region represents the bootstrap-derived 95% confidence interval for the smoothed curve. Red circles denote grouped calibration summaries based on 5 approximately equal-sized risk strata, plotted as mean predicted probability versus observed event rate; vertical error bars indicate exact 95% Clopper-Pearson confidence intervals for the observed event rate within each group. Overall calibration metrics are shown in the inset, including the Brier score, calibration intercept, and calibration slope.
